# The Impact of Waiting Times on Behavioral Outcomes for Children with Otitis Media: Results from an Urban Ear, Nose, and Throat Telehealth Service

**DOI:** 10.1089/tmr.2023.0055

**Published:** 2023-12-08

**Authors:** Ali A.H. Altamimi, Monique Robinson, Eman M.A. Alenezi, Jafri Kuthubutheen, Tamara Veselinović, Greta Bernabei, Tanisha Cayley, Robyn S.M. Choi, Christopher G. Brennan-Jones

**Affiliations:** ^1^ Telethon Kids Institute, The University of Western Australia, Perth, Australia.; ^2^ Medical School, The University of Western Australia, Perth, Australia.; ^3^ Faculty of Life Sciences, Kuwait University, Kuwait City, Kuwait.; ^4^ Faculty of Allied Health Sciences, Kuwait University, Kuwait City, Kuwait.; ^5^ Perth Children's Hospital, Perth, Australia.; ^6^ Department of Audiology, School of Human Sciences, The University of Western Australia, Perth, Australia.; ^7^ School of Allied Health, Faculty of Health Sciences, Curtin University, Perth, Australia.

**Keywords:** otitis media, behavioral outcomes, telehealth, delayed access

## Abstract

**Aim:**

Children with otitis media (OM) experience long waiting times to access Australia's public hospitals due to limited capacity. The aim of this article is to utilize an Ear, Nose, and Throat (ENT) telehealth service (the Ear Portal) to examine whether delayed access to specialist care is associated with poorer behavioral outcomes for children with OM.

**Methods:**

Participants in the study included 45 children who were referred to ENT specialists due to recurrent and persistent OM. Children were triaged as semiurgent with a target time-to-assessment of 90 days or nonurgent with a target time-to-assessment of 365 days. The behavioral outcomes of children were assessed using the parent report Strengths and Difficulties Questionnaire (SDQ). Descriptive statistics and adjusted multiple linear regression models were used to compare children who received access to the service within the time-to-assessment target of their triage category (“on-boundary”; *n* = 17) and outside the time-to-assessment target (“off-boundary”; *n* = 28). Spearman correlation analysis was used to explore the relationship between the internalizing, externalizing, and total SDQ scores as a function of waiting times in days.

**Results:**

Borderline or abnormal SDQ scores ranged from 24.4% to 42.2% across the study participants. The regression analysis showed a statistically significant association between the off-boundary group and higher scores (i.e., poorer) on the peer, emotional, conduct, internalizing, and total problems subscales. Further, lengthy waiting times were significantly correlated with higher internalizing problems. These findings indicate that longer waiting times may lead to poorer behavioral outcomes for children with OM.

Clinical Trial Registration: (ACTRN1269000039189p).

**Conclusion:**

Children with recurrent and persistent OM referred to ENT outpatient care were found to have significantly more behavioral difficulties if their waiting times exceeded the recommended timeframes for their triaged referrals. Additionally, they experienced more internalizing problems that correlated with longer waiting times. This highlights the calls for alterations in current clinical practice given the lengthy waiting times in Australia's public hospitals.

## Introduction

Otitis media (OM) is an inflammatory condition affecting the middle ear and encompasses both acute OM (AOM) and OM with effusion (OME).^1^ AOM typically follows a viral upper respiratory tract infection, causing symptoms like pain and fever, while OME is often asymptomatic. Both types can be associated with middle ear effusion which may lead to varying degrees of conductive hearing loss. Despite the high rates of spontaneous resolution, a subset of children can experience recurrent and persistent OM, leading to prolonged suffering from OM-related symptoms that can impact their quality of life and functional health status.^2^

It is thought that prolonged OM-related hearing loss in the early years of life may interfere with children's developmental outcomes.^3,4^ This may have potential consequences on their auditory processing skills, language development, and educational achievement,^5,6^ which may ultimately impact their behavioral outcomes. Children with a history of recurrent and persistent OM with or without ventilation tube insertion (the primary surgical procedure for OM) have been found to experience a wide range of behavioral problems that can persist into early adolescent years.^7,8^ Examples of behavioral problems include social and emotional difficulties, increased anxiety, as well as attention and hyperactivity problems.^7,9^

Management of recurrent and persistent OM typically involves interventions from Ear, Nose, and Throat (ENT) specialists. In Australia, publicly funded, tertiary-level ENT outpatient care can be accessed for no charge to patients at the point of care but requires a referral from a primary health care provider. Referrals for ENT services from primary care providers are triaged by the tertiary hospital staff (typically ENT specialists) into categories of urgency to ensure the provision of care within specific target timeframes based on the anticipated severity of illness and other relevant factors.^10^ However, adherence to the recommended timeframes is a major challenge for tertiary ENT departments across Australia. This is due to a variety of reasons, including the increasing demand for Australia's public hospital outpatient clinics and the outbreak of COVID-19, which has exacerbated the existing challenges hospitals face in trying to provide care within the recommended waiting times.^11,12^

Tertiary-level ENT departments also need to allocate enough resources to provide appointments for children requiring urgent care. As urgent care is a high priority, children referred with OM (who typically will not meet the criteria for urgent care) can endure prolonged waiting times for assessment and treatment that can be up to 2 years or more. If surgical intervention is recommended, children will endure an additional waiting time for surgical intervention.^13^ Inherently, this increases the likelihood of prolonged exposure to preventable conductive hearing loss.

Despite this, little is known about whether lengthy waiting periods of unresolved OM can have an influence on children's behavioral development. This study sets out to address this gap by utilizing an existing hospital-based telehealth service to examine whether long waiting times to receive ENT specialist care can impact behavioral outcomes in children with recurrent and persistent OM. This will inform current clinical practice on the potential impact of delayed intervention for children with OM on behavioral development and identify the potential role of telehealth in the provision of timely care.

## Methods

### Participants

The current study is part of the Ear Portal study, a prospective telehealth service designed to facilitate early access to ENT outpatient care. Children were identified by routine monitoring of the Perth Children's Hospital (PCH) ENT outpatient waiting list and involved children triaged as category 2 and category 3. Children triaged as category 2 are deemed “semiurgent” and are expected to receive specialist care within 90 days of their ENT referral, while category 3 is classified as “nonurgent” with an expected timeframe of 365 days to receive care.

Caregivers were contacted by the Ear Portal research assistant (RA) to ascertain interest in participation, confirm eligibility, and obtain verbal consent. A signed informed consent was also obtained on the day of the appointment, which was scheduled based on the caregivers' preference. Screening appointments included a comprehensive in-person evaluation of middle ear health and were conducted by a trained RA or an audiologist. Subsequently, the Ear Portal multidisciplinary team, consisting of an ENT specialist, an ENT clinical nurse specialist, and an audiologist, provided asynchronous individualized care plans that were delivered to caregivers by the study RA via phone calls. A complete description of the Ear Portal procedures has been detailed elsewhere.^14^

### Inclusion criteria

Children were eligible to participate in the current study if they had been referred to PCH ENT outpatient care due to recurrent and persistent OM, had not received care from a private ENT clinic, resided within 60 km of the greater metropolitan area of Perth, Western Australia, and were between the ages of 3 and 11 years old.

Children were not eligible to participate in this study if they were triaged as category 1 (i.e., requiring urgent care), had a complex medical history (e.g., craniofacial abnormalities) that required face-to-face ENT appointments, and had been diagnosed with a condition affecting behavioral development (e.g., Autism Spectrum Disorder).

### Outcome measures

The current study administered the parent report Strengths and Difficulties Questionnaire (SDQ) to evaluate the psychological well-being of the children since August 2022. The SDQ is a widely used questionnaire and has been shown to have sound psychometric properties.^15^ It consists of four subscales measuring emotional, peer, attention/hyperactivity, and conduct problems, along with a prosocial subscale highlighting behavioral strengths. Each subscale is scored based on a 3-point scale ranging from 0 to 2, with higher scores indicative of more behavioral difficulties. Summing the SDQ subscales (except for the prosocial subscale) generates a total score. Additionally, internalizing problems can be generated by summing the scores of the emotional and peer problems subscales, while summing the scores of the attention/hyperactivity and conduct problems subscales generated externalizing problems.

### Statistical analysis

Descriptive statistics were used to summarize the sociodemographic characteristics of the study participants. A binary variable was created to categorize children into the on-boundary and the off-boundary groups. Children were categorized into these groups based on the expected intervention timeframes of their triage categories and the time they accessed the service (i.e., within or after the recommended timeframes). The frequency and percentages were calculated for children who had scores falling outside the normal range of the SDQ (i.e., borderline and abnormal) for both groups.

Multiple linear regression models were then used to explore if the timing of access to the service predicts scores on all SDQ subscales, in addition to the internalizing, externalizing, and total problem scales. All regression analyses were controlled for age, gender, and ethnicity. The Spearman correlation analysis was conducted to investigate the change in scores of the internalizing and externalizing SDQ scores in relation to waiting times as a continuous variable. For all analyses, a *p*-value of ≤0.05 was considered statistically significant. The data were analyzed using SPSS software version 29.0.1.0.

### Ethical consideration

Ethical approvals for the Ear Portal project were obtained from the Child and Adolescent Ethical Committee, the Western Australian Aboriginal Health Ethics Committee, and the University of Western Australia. The study was prospectively registered on the Australian New Zealand Clinical Trials Registry (ACTRN1269000039189p).

## Results

A total of 45 participants were included in this study. Of those, 17 (37.7%) participants received access to the service within the recommended timeframes according to their triaged categories (on-boundary group) and had a mean age of 6.7 years (standard deviation [SD] = 2.7). In contrast, 28 (62.3%) participants received access to the service after the recommended timeframes according to their triaged categories (off-boundary group) and had a mean age of 5.9 (SD = 2.4). Exploratory analysis showed a mean of 256.8 days (SD = 158.5, range = 6–743), which reflect the additional waiting times children in the off-boundary group experienced beyond the recommended timeframes for their triaged categories.

Chi-square analysis revealed no significant differences in the sociodemographic variables between the two groups (Table 1). In general, the mean scores of the off-boundary group were higher (i.e., poorer) compared to the on-boundary group with more children in the off-boundary group having scores falling outside the normal range of the SDQ subscales (Table 2).

**Table 1.** Sociodemographic Variables of the Study Participants

Characteristics	On-boundary**, *n*** = 17^a^	Off-boundary, ***n*** = 28^a^	***p*** ^b^
Age	6.7 (2.7)	5.9 (2.4)	0.14
Gender			
Males	41.2 (7)	50.0 (14)	
Females	58.8 (10)	50.0 (14)	0.56
Ethnicity			
Caucasian	73.3 (12)	70.6 (21)	
Other	26.7 (5)	29.4 (7)	0.74
Mother completed year 12^c^			
Yes	82.4 (14)	80.8 (21)	
No	17.6 (3)	19.2 (5)	0.89
Father completed year 12^c^			
Yes	75.0 (12)	68.0 (17)	
No	25.0 (4)	32.0 (8)	0.63
Exposure to passive smoking^c^			
Yes	11.8 (2)	17.8 (8)	
No	88.2 (15)	42.2 (19)	0.28

^a^ Indicates mean (standard deviation) or % (*n*).

^b^ Pearson's chi-squared test or Fisher's exact test.

^c^ Contain missing items.

**Table 2.** The Frequency and Percentages of Children Who Had Scores Falling Outside the Normal Range of the Strengths and Difficulties Questionnaire for Both Groups

SDQ subscales	On-boundary (***n*** = 17), % (***n***)	Off-boundary (***n*** = 28), % (***n***)	All, % (***n***)
Emotional	17.6% (3)	57.1% (16)	42.2% (22)
Conduct	11.8% (2)	42.9% (12)	31.1% (14)
Hyperactivity	129.4% (5)	39.3% (11)	35.6% (16)
Peer	5.9% (1)	60.7% (17)	40.0% (18)
Internalizing	5.9% (1)	35.7% (10)	24.4% (11)
Externalizing	11.8% (2)	35.7% (10)	26.7% (12)
Total	29.4 (5)	51.9% (14)	43.2% (19)

On-boundary: Accessed the service within the recommended timeframes of triaged categories.

Off-boundary: Accessed the service beyond the recommended timeframes of triaged categories.

SDQ, Strengths and Difficulties Questionnaire.

**FIG. 1.**
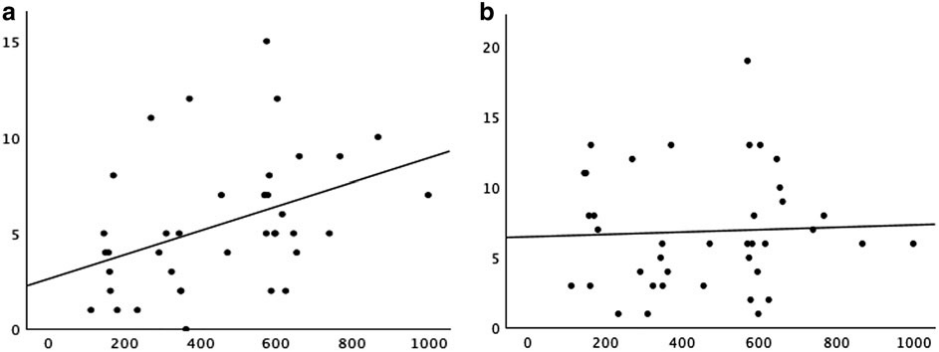
A scatterplot with Fitted Line shows the distribution of raw internalizing **(a)** and externalizing **(b)** scores of children in Category 3 (*n* = 39) as a function of waiting times. *X*-axis: waiting time (days). *Y*-axis: internalizing **(a)** and externalizing **(b)** raw scores.

In the linear regression models that compared SDQ scores between the groups, it was revealed that the off-boundary group exhibited significantly greater difficulties in the peer, conduct, internalizing, and total subscales (Table 3). However, the hyperactivity and the prosocial subscales scores were not statistically different between the groups. The Spearman correlation analysis revealed a moderate, positive relationship between waiting times in days and increased internalizing scores for children triaged to Category 3 (*r* = 0.44, *p* = 0.005) (Fig. 1). A positive relationship was also found for the total (*r* = 0.22) and externalizing scores (*r* = 0.07); however, the results were not statistically significant (*p* > 0.05). The Spearman correlation analysis was not conducted for children in Category 2 due to the small sample size.

**Table 3.** Multiple Linear Regression Comparing the Effects of Receiving Access to Care After the Recommended Timeframes of Triage Categories

SDQ subscales	***b*** (SE)	***p***	95% CI
Emotional	1.4 (0.8)	0.06	−0.1 to 2.8
Peer	1.9 (0.5)	**<0.001** ^*^	0.9 to 2.9
Conduct	1.3 (0.7)	**0.06**	−0.1 to 2.6
Hyperactivity	0.6 (0.8)	0.43	−1.0 to 2.2
Prosocial	−0.1 (4.3)	0.76	−1.0 to 0.7
Internalizing	3.3 (1.9)	**<0.001** ^*^	1.4 to 5.2
Externalizing	1.9 (1.3)	0.14	−0.7 to 4.7
Total	5.1 (1.9)	**0.013** ^*^	1.2 to 9.1

All analyses were controlled for age, gender, and ethnicity. Bold and asterisk indicates statistical significance.

*b* (SE), unstandardized coefficients (standard error); CI, confidence interval.

## Discussion

This study examined the behavioral outcomes in children with recurrent or persistent OM awaiting ENT specialist intervention by utilizing a hospital-based telehealth service. Overall, there was a high incidence of borderline and abnormal SDQ scores across the study participants. Additionally, receiving access to the service after the recommended timeframes for triaged categories resulted in significantly more behavioral difficulties, which correlated with the longer duration of wait times.

The frequency of abnormal SDQ scores displayed variability within this cohort, with emotional (42.2%) and peer (40%) difficulties being the most commonly reported problems. Moreover, children in the off-boundary group exhibited significantly higher peer, internalizing, and total problem scores compared to the on-boundary group. These findings suggest that the timing of intervention may have an influence on the manifestation of behavioral problems for children with recurrent and persistent OM, which is a factor not often explored in prior studies.

The relationship between the delayed intervention of OM and behavioral development may be explained by the intricate dynamics of each factor and the possible interaction between them over time. The associated symptoms may significantly alter their mood and have a prolonged adverse impact on their quality of life and daily life activities (e.g., school absenteeism, activity limitations).^2^ This impact may be further exacerbated if language development is not progressing adequately. Therefore, the potential interaction of these factors, in addition to parental stress and social factors,^16^ could have a significant influence on children's behavioral development, particularly when these factors are prolonged due to delayed necessary interventions.

The impact of intervention time for OM on behavioral development is further supported by the results of the correlation analysis, which revealed a moderately positive association between the internalizing problems subscale and the passage of time. That is, as the duration of waiting time for receiving care increases, there is a corresponding increase in internalizing problem scores for children with OM. Internalizing behavioral problems are commonly reported among children with hearing loss.^17^ Thus, it is plausible that children who encountered lengthy waiting times experienced protracted periods of OM-related hearing impairment, ultimately impacting their behavioral development.

When comparing these findings with the findings of previous studies, several authors have reported a link between recurrent OM in early childhood and later behavioral and internalizing problems in children with or without ventilation tube insertion.^7,8^ Additionally, children with a history of recurrent OM were found to be at an increased risk of later diagnoses of various mental health and developmental delays.^18^ The scarcity of information on intervention time in prior studies makes it challenging to predict whether delayed intervention was a contributing factor to the observed behavioral difficulties. However, given that children in the on-boundary group did not exhibit significant behavioral difficulties compared to the off-boundary group, this may suggest a possible interplay between waiting times and other factors that need to be simultaneously considered during the provision of ENT specialist care.

Children with OM awaiting ENT outpatient care may be at a disadvantage in the triage process since this is primarily based on clinical symptoms disclosed in their referrals. As a consequence, any OM-related behavioral difficulties that may have emerged during the waiting period can be inadvertently overlooked unless specific concerns are raised by their caregivers to the referring practitioner. Unlike externalizing problems, internalizing problems are more subtle and generally difficult to identify in younger children,^19^ placing added responsibilities on caregivers to have informed concepts of age-appropriate behavioral milestones. Internalizing problems such as anxiety and depression constitute a high burden of disease in children and adults.^20^ They can also have a considerable impact on identity development, and social and emotional functioning.^21^ Moreover, untreated or unmanaged behavioral difficulties in early childhood can significantly impact children's educational attainment and increase the economic burden.^17,22^ Therefore, the identification of potential contributors to behavioral difficulties in early childhood is of utmost importance and may require the adaptation of alternative care delivery pathways (e.g., telehealth services) that may enable timely access to ENT specialist care.

Studies examining the feasibility of ENT telehealth services have demonstrated promising results, highlighting their potential to reduce waiting times and providing timely access to care for children with OM.^14,23,24^ Hence, the implementation of telehealth review as part of the advanced triage of patients in ENT departments with long waiting lists may serve as a feasible approach that may enable regular, prompt, and comprehensive monitoring of recurrent and persistent OM. This would help facilitate early identification and intervention of children at risk of behavioral problems who are currently waiting for assessment. This may also indirectly alleviate the strain on ENT specialists by reducing the rate of OM cases being waitlisted that may not require surgical intervention but may benefit from other therapeutic interventions.

### Strengths, limitations, and future directions

The study design underlined the need to continue efforts to address wait times that exceed the recommended timeframes to ensure negative developmental outcomes are not generated by failure to act. It is acknowledged that a number of factors limited the findings of this study, including the small sample size and the absence of important baseline data (e.g., SDQ scores and hearing levels). This introduces uncertainties regarding the degree of association between delayed access to care and behavioral outcomes for children with OM. However, it is promising to observe significantly fewer behavioral problems when timely access to care is provided. Additionally, ∼13.3% (*n* = 6) of children were triaged as category 2 (i.e., more urgent compared to Category 3), which did not allow us to explore the relationship between SDQ scores and waiting times for this category and may therefore underestimate the findings of this study.

The categorization of children into on-and-off boundaries was utilized in this study to reflect real-life circumstances that children may experience in public hospitals. Therefore, the absence or emergence of behavioral difficulties should not hinge solely on whether children receive care pre- or -post the recommended timeframes, especially since developmental outcomes are typically not factored into the triage process.

Future studies with larger sample sizes are warranted to replicate the findings of this study and to explore whether long-term behavioral difficulties can be mitigated by the provision of timely care for OM via telehealth or other services that can provide additional support or expedite care for children placed on waiting lists for ENT services. Additionally, examining the feasibility of hospital-based telehealth services to be economically viable, while providing timely access to care for children with OM can facilitate the integration of these services into mainstream practice.

## Conclusions

Children with recurrent and persistent OM awaiting ENT specialist intervention at a tertiary hospital exhibited significant behavioral difficulties. The impact was significantly higher in children who had access to the ENT telehealth service after the recommended timeframes for their triaged referrals compared to those who received earlier access to the service. The study's findings suggest that lengthy waiting times can potentially contribute to the manifestation of behavioral problems in children with untreated OM.

## Abbreviations Used

AOM: acute otitis media
ENT: Ear, Nose, and Throat
OM: otitis media
OME: OM with effusion
PCH: Perth Children's Hospital
RA: research assistant
SDQ: Strengths and Difficulties Questionnaire
